# Fine-scale mapping of irrigation suitability in South Africa using ensemble modelling

**DOI:** 10.1038/s41598-025-12820-y

**Published:** 2025-10-21

**Authors:** Kudzai S. Mpakairi, Timothy Dube, Mbulisi Sibanda, Onisimo Mutanga, Luxon Nhamo, Sylvester Mpandeli

**Affiliations:** 1 Institute for Water Studies, Faculty of Natural Sciences, University of the Western Cape, Cape Town, South Africa; 2 Department of Geography, Environmental Studies and Tourism, University of the Western Cape, Cape Town, South Africa; 3 Discipline of Geography and Environmental Science, University of KwaZulu-Natal, Pietermaritzburg, South Africa; 4 Water Research Commission of South Africa, Lynwood Manor, Pretoria, 0081 South Africa; 5 Centre for Transformative Agricultural and Food Systems (CTAFS), School of Agricultural, Earth and Environmental Sciences, University of KwaZulu-Natal, Pietermaritzburg, 3209 South Africa; 6 Department of Environmental, Water and Earth Sciences, Tshwane University of Technology (TUT), Pretoria, South Africa

**Keywords:** Agriculture potential areas, Climate change, Croplands, Machine learning, Food security, Sustainable agriculture, Agroecology, Biodiversity, Ecological modelling, Environmental sciences

## Abstract

Food insecurity, exacerbated by a growing population and environmental change, poses a significant challenge in Southern Africa. Enhancing agricultural productivity through efficient irrigation practices is crucial for achieving food and water security and sustainable development goals. This study applied an ensemble modelling approach to identify and assess irrigation suitability areas across South Africa, combining the predictive power of Random Forest, Extreme Gradient Boosting (XGBoost), and Gradient Boosting Machine (GBM) algorithms. These machine learning models were applied using cropland presence/pseudo-absence data and a suite of predictor variables. The ensemble model, leveraging a weighted averaging approach based on individual model performance, outperformed the individual models, achieving a TSS of 0.66 and an AUC of 0.90. Land use, population density, and elevation were identified as key factors determining irrigation suitability. The ensemble model also revealed substantial spatial variation in irrigation potential across South Africa, with the Northern Cape and Western Cape provinces exhibiting the largest suitable areas. The results provide critical information for targeted irrigation development, enabling efficient resource allocation, and maximising agricultural productivity. This data-driven approach offers a robust framework for sustainable agrarian planning in the face of increasing food demands and climate change, contributing to enhanced food security and economic development in South Africa.

## Introduction

Due to rapid population growth, southern Africa is faced with increasing challenges related to food insecurity^1,2^. To avert a potential crisis, it is crucial to enhance agricultural productivity, a critical factor in achieving Sustainable Development Goals (SDGs) 1 and 2, advocating for ending hunger and abetting poverty^3,4^. However, improving agricultural output is complicated by the region’s highly variable climate and diverse soil conditions as well as poor farming management practices^5,6^. For instance, South Africa’s climate ranges from arid to subtropical, making agricultural planning complex^7^. The country depends heavily on irrigated agriculture to support export-oriented production such as sugar cane and fruit as well as for domestic food needs^8,9^. South Africa could focus on expanding its area under irrigation farming to enhance food security since irrigation redresses food insecurity at national and household levels^9,10^. This expansion, however, must be guided by data-driven models that accurately predict suitable irrigation zones. Expanding the irrigated area to suitable areas facilitates efficient resource use, mitigates environmental degradation from the overuse of nutrients and chemicals and reduces water allocated to agriculture as the sector already accounts for over 60% of freshwater consumption in South Africa^10,11^.

Traditional irrigation suitability assessments often rely on land capability classifications, considering soil texture, slope or terrain, and general water availability^12–14^. However, these methods can be time-consuming and depend on localised ground-based measurements, often failing to capture environmental variables’ spatial heterogeneity and complex interactions^14–16^. While tools such as the Analytical Hierarchy Process (AHP) offer a more structured approach by incorporating environmental determinants (e.g., topography, soil characteristics, and broad water availability) and ranking areas based on irrigation potential, they still assume hierarchical decision-making and may not adequately represent non-linear interactions within complex ecosystems^14,15,17^. Unlike land capability classifications and AHP, machine learning (ML) algorithms, including support vector machines, random forests, maximum entropy models (MaxEnt), and boosted regression trees, can integrate large, diverse datasets and capture linear and non-linear interactions between variables, leading to more robust and accurate predictions^18,19^. Studies done in Benin and Togo^20^, Tunisia^21^ and Ghana^18^ demonstrated the capabilities of ML and how it can leverage a wider range of environmental variables (e.g., temperature, rainfall, soil pH, elevation) and discern complex spatial and ecological relationships relevant to crop suitability.

Another compelling approach within machine learning is the use of ensemble modelling. Ensemble methods combine the predictions of multiple individual models (often called “base learners”) to create an accurate prediction^21,22^. The underlying principle is that different models may capture various aspects of the complex relationships within the data, and by combining their strengths, the ensemble can achieve higher predictive accuracy and robustness than any specific model^23–25^. Ensemble modelling has been highly effective in various domains, including species distribution modelling^24,26^, water quality assessment^27,28^, and land cover classification^29,30^. However, its application in predicting irrigation suitability remains limited. By harnessing the power of ensemble modelling, researchers and policymakers can continuously update existing maps and create more accurate, reliable representations of irrigation suitability, fostering more informed and sustainable agricultural development^31^. These models allow for integrating large and diverse datasets and provide robust predictions that account for linear and non-linear relationships between the various variables^32^. By ensemble modelling, researchers and policymakers can better inform sustainable and productive agricultural expansion.

Commonly used environmental determinants for irrigation suitability have primarily been bioclimatic variables such as temperature, precipitation, and evapotranspiration, with minimal inclusion of other factors such as groundwater availability, market access, and infrastructure development^18,33,34^. Groundwater depth or storage is often excluded from suitability models due to data constraints and the complexity of modelling groundwater systems, especially in heterogeneous environments^35^. However, the inclusion of groundwater data for water-scarce countries such as South Africa is imperative, since ongoing irrigation practices depend on groundwater resources particularly in regions such as Limpopo, where surface water resources are limited^36,37^. Incorporating groundwater into ensemble models could enhance its accuracy and reflect the feasibility of agriculture expansion in these arid environments.

This study uses an ensemble of machine learning algorithms to predict, at a national scale, the areas most suitable for irrigated agriculture in South Africa. The environmental determinants considered in this study include vital factors relevant to the country’s agricultural landscape, such as groundwater availability, which have been neglected in most studies. Predicting the suitability of irrigation farming is essential for optimising land use, increasing agricultural productivity, and enhancing food security in South Africa. By providing insights into the spatial distribution of irrigation potential, this research offers valuable guidance to policymakers, community of practice and other stakeholders, enabling informed decisions about irrigation expansion. Ultimately, this study contributes towards promoting sustainable agricultural practices that enhance food security and minimise environmental impacts, contributing to long-term agrarian resilience in the region.

## Methods and materials

### Study area

**Fig. 1:**
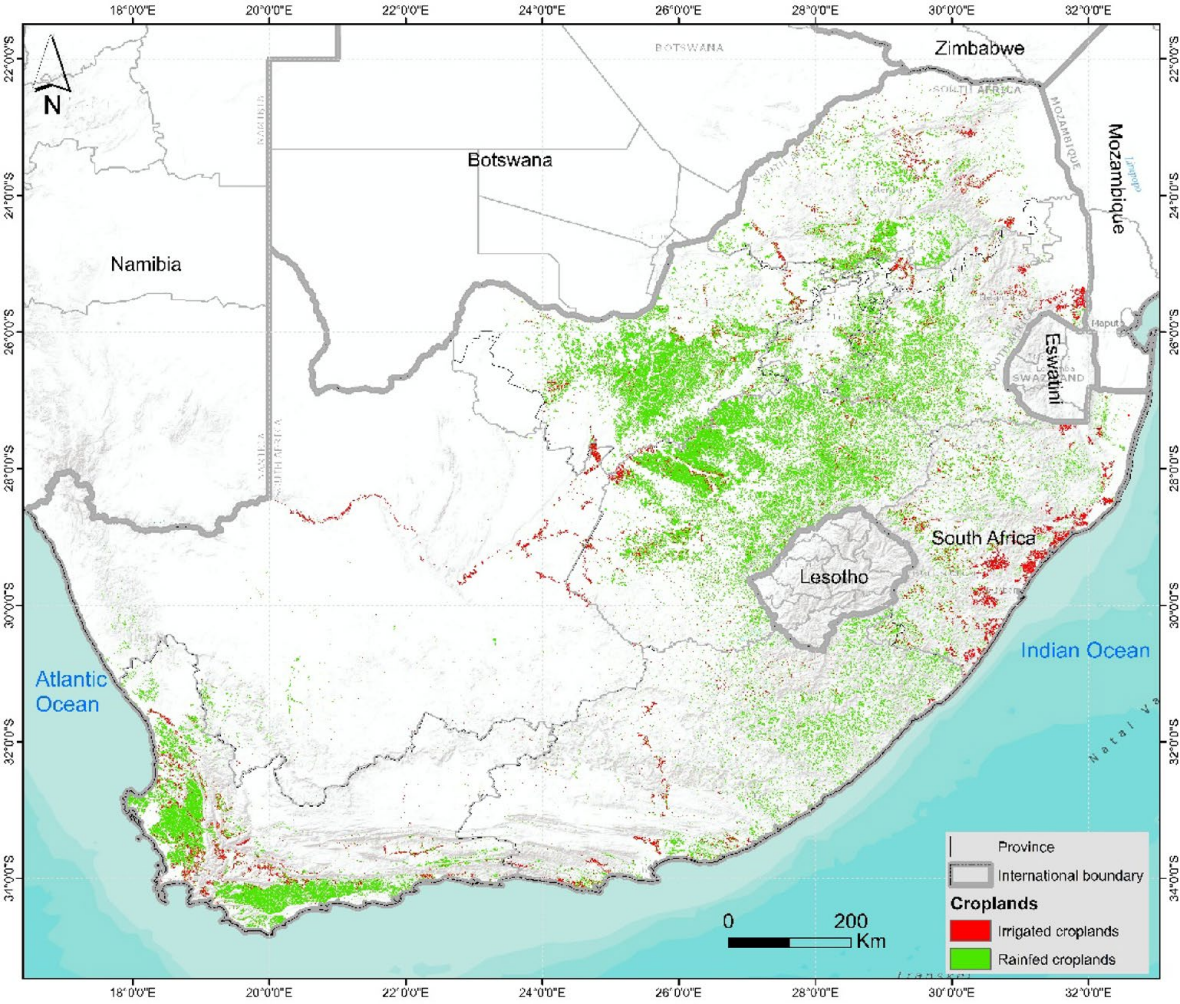
Map of the distribution of irrigated and rainfed croplands in South Africa^41^.

The study focused on South Africa, which exhibits remarkable geographic and climatic diversity, stretching from the western regions’ arid deserts to the moist east’s subtropical climates (Fig. 1). The distinct topography, varying altitudes, and proximity to the Atlantic and Indian Oceans contribute to a highly varied environment across its nine provinces^38,39^. The climatic variations influence the existence of several biomes, including savannas, grasslands, deserts, and Mediterranean-like regions in the southwestern Cape, each presenting unique conditions for agriculture^39,40^.

The agricultural landscape in South Africa is varied, featuring diverse activities such as grain cultivation, sugar production, forestry, vegetable and fruit farming, livestock rearing, and subsistence farming^42,43^. Mpakairi, Dube^41^ estimated that 40% of irrigated croplands in South Africa were concentrated in Free State, Northwest, and Western Cape provinces. These regions also host most commercial agricultural activities, whereas subsistence farming predominates in provinces such as KwaZulu Natal and Eastern Cape^44,45^. Due to the country’s largely semi-arid climate, irrigation is crucial in sustaining agriculture, particularly in areas where natural rainfall is insufficient or too variable to support crop production^42,43^.

### Data collection

#### Irrigation presence data

The irrigation presence data identifying irrigated (*n* = 121,830) croplands were provided by Mpakairi, Dube^41^. The data were derived from an integrated approach using a random forest classifier and deep neural network. The random forest model was used to classify South Africa into different land cover types, including croplands, and achieved an overall accuracy of 0.77. The cropland extent identified by the random forest model was then reclassified into irrigated and rainfed croplands through a deep learning algorithm, which had an overall accuracy of 0.71. As this product underwent a comprehensive peer-reviewed validation, no additional assessment was performed for this study. More details on the methodology can be found in Mpakairi, Dube^41^. The irrigation data were split into training (n = 97,464) and test (n = 24,366) data. The training data were used for model calibration, and the test data were used for model evaluation. The large volume of presence points ensured sufficient data to support the complexity of ensemble models without overfitting. Although classical power analysis is not standard practice in machine learning applications, this sample size compares favourably with similar national-scale modelling efforts, thereby supporting model robustness^46–48^.

#### Pseudo-absence data

To enhance model accuracy and ensure a balanced prediction of irrigation suitability, pseudo-absence data were incorporated into the modelling process^49,50^. In this study, pseudo-absence data represented areas where irrigation is unlikely to occur (e.g., nature reserves, national parks and water bodies), providing negative examples that the models can learn from in addition to the actual irrigation presence data^50^. This approach is critical for improving the generalisation of the models and avoiding bias toward sampled areas (i.e., areas with irrigation)^51^.

To create the pseudo-absence dataset, the BIOMOD package in R was utilised^52^. Pseudo-absence points were randomly selected from areas without irrigated croplands. The number of pseudo-absence points was set at 5,000 for each model run, and the random sampling process was repeated twice to ensure variability in the selection of absence points. This strategy contributed to creating a balanced dataset that accurately represented both suitable and non-suitable areas for irrigation, which was then used to train the models and improve prediction reliability.

#### Environmental variables

To predict the distribution of the irrigated areas in South Africa, the study used a suite of twelve (n = 12) environmental variables, as shown in Table 1. The variables represented different ecological components, including climate, soil, infrastructure, hydrological, elevation, land use and groundwater. These variables were selected owing to their fundamental and well-documented role in influencing crop growth and development.

**Table 1:** List of the environmental variables used in the study for 2023 alone.

Variables group	Variable	Spatial resolution	Temporal resolution	Source
Climate	Mean temperature (°C)	~ 9 km	Daily aggregated	ERA5-Land
	Potential Evapotranspiration (mm)	500 m	8-day	Moderate Resolution Imaging Spectroradiometer (MODIS)
	Annual precipitation (mm)	~ 9 km	Daily aggregated	ERA5-Land
Soil	Soil texture (0–20 cm depth)	250 m	–	SoilGrids
	Percent soil moisture content (33 KPA)	250 m	–	SoilGrids
Economic factor	Population density (people per square kilometre)	~ 1 km	Annual	Center for International Earth Science Information Network (CIESIN)
Hydrological	Distance to rivers (m)	30 m	–	Open Street Map
Elevation & terrain	Elevation (resampled)	30 m	–	NASA Shuttle Radar Topography Mission (SRTM)
	Terrain slope (°)	30 m	–	NASA SRTM
Land use	Land cover/land	10 m	Annual	ESRI
Groundwater variables	Groundwater depth (m)	1 km	–	GLOBGM
	Groundwater storage (centimetres of equivalent water thickness)	~ 55 km	Annual	Gravity Recovery and Climate Experiment

#### Climate and soil variables

Mean temperature and annual precipitation data were sourced from the European Centre for Medium-Range Weather Forecasts (ECMWF) ERA-5 Land dataset, a widely recognised and extensively validated reanalysis dataset providing high-resolution global coverage of land surface variables^53,54^. ERA-5 Land offers significant improvements over previous reanalysis products, incorporating more sophisticated modelling techniques and a wider range of observational data, enhancing accuracy and reliability^54^. Potential evapotranspiration (PET) data, a crucial indicator of atmospheric water demand and a key driver of irrigation requirements were derived from the Moderate Resolution Imaging Spectroradiometer (MODIS) Global Evapotranspiration Project (MOD16). MOD16 provides valuable estimates of ET globally, offering a spatially explicit representation of water fluxes^55–57^. These climate and environmental variables *vis;* temperature, precipitation, and evapotranspiration were specifically chosen due to their fundamental and well-documented role in influencing crop growth and development, thus serving as key factors in evaluating irrigation requirements^58–60^. Temperature directly affects plant physiological processes, including photosynthesis and respiration^61^, while precipitation provides the primary water source for plant uptake^62^. PET, representing the combined loss of water from the land surface through evaporation and transpiration, is a critical parameter for determining crops’ water balance and irrigation needs^63^.

To ensure temporal relevance and capture the most recent climatic conditions, the mean values for potential evapotranspiration, temperature, and precipitation were computed exclusively using data from the year 2023. Focusing on a single year allowed for a more contemporary assessment of irrigation suitability, reflecting current climate patterns and potentially avoiding biases introduced by averaging over extended periods that might include significant climatic variability. While multi-year averages can provide a broader picture of climate, using a single year’s data can be particularly relevant for short-term agricultural planning and decision-making^64^.

Soil characteristics, including texture and moisture content, were incorporated into the analysis due to their significant influence on irrigation feasibility^65,66^. Soil texture, which refers to the proportion of sand, silt, and clay particles in the soil, affects water holding capacity, drainage, and aeration, which are crucial for plant growth and irrigation management^67,68^. The soil texture included clay, silty clay, sandy clay, clay loam, silty clay loam, sandy clay loam, loam, silt loam, sandy loam, silt and loamy sand. Soil moisture content, representing the amount of water in the soil, directly influences water availability to plants and is a critical factor in determining irrigation needs^69,70^. Accurate representation of these soil properties is essential for developing realistic and reliable irrigation suitability assessments.

However, soil properties such as organic carbon and nitrogen content were intentionally excluded from consideration in this initial modelling effort. While these factors are crucial for overall soil fertility and crop productivity, their inclusion can introduce complexities and limit the model’s generalizability across various crops and diverse geographical regions. Organic carbon and nitrogen content can vary significantly depending on land management practices, soil type, and other localised factors^35,71^. By focusing on more fundamental soil properties such as texture and moisture, the model aims to maintain broader applicability and provide a more general assessment of irrigation suitability.

#### Hydrology and economic variables

The rivers network for South Africa was obtained from OpenStreetMap, a freely available and collaboratively edited world map^72^. The Euclidean distance function within the Google Earth Engine (GEE) platform was employed to quantify the proximity to rivers, a crucial factor influencing irrigation feasibility. This variable was incorporated due to the critical role that access to surface water plays in determining the feasibility of irrigation, particularly in regions with limited or inaccessible groundwater resources^35,71^.

Population density data for 2019 to 2021 were sourced from the Centre for International Earth Science Information Network (CIESIN). This demographic variable was included in the analysis to reflect the availability of agricultural labour and proximity to potential markets for agricultural produce^73,74^. A higher population density near irrigable land can indicate a readily available workforce for agricultural activities^75^. Furthermore, population density was used to infer proximity to markets. This is critical since reduced transportation costs for agrarian outputs, enhance the economic viability of irrigated agriculture^75^. In addition, market access is essential for farmers to sell their produce and generate income, a key driver of agricultural development^76^.

However, other potentially influential economic factors, such as land ownership patterns, access to credit, input costs (e.g., fertilisers, seeds), and market prices for agricultural commodities, were deliberately excluded from this analysis. While these factors undoubtedly play a significant role in the overall economic viability and success of irrigation projects, their inclusion at this broad-scale mapping stage could introduce complexities and potentially confound the assessment of general irrigation suitability. These more nuanced economic factors are more relevant for subsequent stages of analysis, specifically for identifying priority areas for the implementation of irrigation projects rather than for the initial broad-scale mapping of general irrigated suitability. The current study focuses on delineating biophysically and hydrologically suitable areas for irrigation, providing a foundational layer of information upon which more detailed economic analyses can be built.

#### Elevation and slope variables

Elevation and slope data were obtained from the Shuttle Radar Topography Mission (SRTM), a near-global digital elevation model (DEM) providing high-resolution topographical information. Elevation plays a multifaceted role in influencing irrigation suitability. Elevation directly affects water flow and drainage patterns^77^. Lower elevations, generally characterised by flatter terrain, tend to be more suitable for irrigation due to reduced runoff and improved water retention^78^. Conversely, higher elevations are often associated with steeper slopes, leading to increased runoff and reduced water infiltration. Subsequently, such areas are not conducive for irrigation agricultural practices^79,80^.

#### Landcover and groundwater variables

Annual land cover data were accessed from the ESRI Living Atlas, a comprehensive repository of global geospatial information, and provided at a high spatial resolution of 10 m. The land cover types include water, trees, flooded vegetation, croplands, built-up, bare ground, snow, cloud and rangelands. However, this study excluded most land-use types except for croplands, bare ground and rangelands since this data are crucial for identifying regions already under cultivation or areas where new irrigation infrastructure could be strategically developed^80–82^. Groundwater depth and storage data, critical factors in assessing the feasibility of irrigation, particularly in areas where surface water resources are scarce, were collected from global groundwater datasets provided by the Global Groundwater Model (GLOBGM) and the Gravity Recovery and Climate Experiment (GRACE). GLOBGM is a global-scale hydrological model that estimates groundwater storage and recharge^83^. At the same time, GRACE is a satellite mission that measures variations in Earth’s gravity field, which can be used to infer changes in groundwater storage^84,85^. In regions with shallow groundwater tables, groundwater can be a readily accessible and cost-effective water source for irrigation^86,87^. Groundwater resources can be vital in arid and semi-arid regions with limited or highly variable surface water availability^88^.

The various datasets were derived at different spatial resolutions and potentially different temporal scales; a rigorous pre-processing step was undertaken to ensure consistency and alignment for the predictive modelling process. For instance, climate data, originally provided at finer temporal resolutions, were aggregated into annual averages to match the temporal scale of the study, focusing on yearly patterns of temperature, precipitation, and evapotranspiration. All predictor datasets were resampled to a common spatial resolution of 1 km to ensure compatibility during modeling. The standardisation of spatial resolution ensured uniformity in the predictive modelling process, allowing for the seamless integration of different datasets and preventing biases due to differing grid sizes. Continuous variables (e.g., climate, elevation) were resampled using bilinear interpolation, while categorical data (e.g., soil type) used nearest-neighbor resampling.

The datasets were drawn from the most recent and validated sources available at the time of the analysis. Although temporal mismatches exist, this approach is consistent with standard practices in spatial suitability and distribution modeling (e.g., Hao, Elith^32^, Mpakairi, Ndaimani^89^, and Huang, An^46^). In such studies, environmental predictors are selected based on their relevance and temporal representativeness. Therefore, the multi-temporal integration used here reflects a practical and widely accepted modeling compromise to maximize data quality and spatial coverage.

### Multicollinearity analysis

Before using the environmental variables for predicting areas suitable for irrigation, multicollinearity analysis was tested on the variables. Multicollinearity is essential in model building, ensuring that highly correlated variables do not bias the model performance^90,91^. Collinear variables can lead to unstable estimates and reduce the interpretability of the model outcomes^91^. The study used a pairwise correlation matrix for all 12 environmental variables to assess multicollinearity. Pearson correlation coefficients (r) were calculated between each pair of variables to identify any significant correlations (r <|0.80|), which indicate high multicollinearity^91,92^. Variables that had a high correlation were excluded from model calibration.

### Ensemble modelling framework

The **Biomod2** package in R was employed to construct the ensemble model for predicting irrigation suitability across South Africa. Biomod2 provides a robust and flexible framework for integrating multiple modelling algorithms, performing cross-validation, optimising hyperparameters, and generating ensemble predictions^52^.

#### Development of individual candidate models

Three machine learning algorithms were used for model development: Random Forest (RF), Extreme Gradient Boosting (XGBoost), and Gradient Boosting Machine (GBM). These algorithms were selected due to their proven ability to capture complex, non-linear relationships between predictor and response variables, essential for accurately predicting irrigation suitability in a diverse environmental context^18,93,94^. These algorithms are widely recognised for their ecological and environmental modelling performance, offering robust and reliable predictions.

RF is particularly effective for handling high-dimensional datasets with complex, non-linear interactions among predictor variables^95,96^. The algorithm operates by constructing decision trees, each trained on a random subset of the data and a random subset of predictor variables^97^. Predictions are generated by aggregating the outputs of individual trees, effectively averaging out noise and reducing overfitting^98^. This approach makes RF robust to noisy or missing data, enhancing its reliability in this study.

GBM is a classical boosting technique that builds weak learners (typically shallow decision trees) and combines their outputs through iterative reweighting to improve predictive performance^99–101^. This algorithm is known for its ability to model intricate relationships and improve prediction accuracy by focusing on correcting the errors made by previous models^100,102^.

XGBoost is a highly efficient and scalable implementation of gradient boosting that introduces regularisation techniques (L1 and L2 penalties) to reduce overfitting, a common issue in complex models^97,103^. Its ability to handle large datasets efficiently, with its robust handling of missing data and regularisation features, makes it particularly suitable for large-scale environmental data applications^104^.

The cropland presence and pseudo-absence data were used as the response variables, while 12 environmental variables (e.g., climate, soil properties, topography) were used as predictors to train the individual models.

#### Cross-validation and model calibration

To ensure the robustness and generalisation of the individual models, we implemented a k-fold cross-validation strategy. Specifically, the dataset was partitioned into two folds, and the process was repeated three times with training and validation data. This repeated partitioning and evaluation procedure provides a more robust estimate of model performance than a single train-test split^105,106^. To address class imbalance due to the lower number of pseudo-absence points (n = 5,000) compared to presence points (n = 121,830), we employed a prevalence adjustment of 0.5 within the Biomod2 framework, following the guidance of Thuiller, Lafourcade^107^. This approach effectively balances the weight assigned to both classes during model calibration and helps to prevent the model from being biased towards the majority class^51,52^.

Hyperparameter optimisation was carried out using the default strategies embedded within Biomod2, which includes an automated search for the best parameter settings to maximise model performance^46^. For Random Forest, we evaluated ntree values of 500, 1000, and 1500, and mtry values of 2, 4, and 6. GBM models were tuned for learning rates (0.01, 0.05, 0.1) and tree depths (3, 5, 7). XGBoost models considered combinations of learning rate (0.01–0.1), maximum depth (3–7), and regularization parameters (lambda, alpha = 0, 1). The optimal parameters, selected based on highest average TSS and AUC-ROC across 10 replicates.

#### Ensemble model

To further enhance prediction accuracy and reduce uncertainty, an ensemble model was created by integrating the outputs of RF, XGBoost, and GBM. Ensemble methods, particularly those combining the strengths of various modelling techniques, have proven to be highly effective in spatial decision-making tasks by leveraging the complementary strengths of individual models^32,89^.

The ensemble model was constructed using a weighted averaging approach, where individual model outputs were weighted according to their respective TSS and AUC-ROC scores. The ensemble model was built using candidate models with a TSS score higher than 0.55. This ensured that the more accurate models predominantly influenced the final predictions through a weighted averaging approach. The weighted averaging approach allowed the ensemble to capitalise on the strengths of the best-performing individual models, resulting in a more reliable and robust irrigation suitability map that integrates the best aspects of machine learning-based predictions.

### Evaluation

The performance of the individual models (GBM, XGBoost, RF) and the ensemble model was rigorously evaluated using the True Skill Statistic (TSS) and the Area Under the Receiver Operating Characteristic Curve (AUC-ROC). The TSS provides a balanced measure of model performance, accounting for sensitivity and specificity^108,109^. At the same time, the AUC-ROC assesses the model’s ability to discriminate between suitable and unsuitable areas, providing a measure of overall predictive power^110,111^. These metrics provided robust benchmarks for comparing individual models and informed the subsequent ensemble creation process^108,110^. While independent spatial validation was not feasible due to data constraints, we applied tenfold cross-validation with spatial stratification to estimate model generalizability.

## Results

### Multicollinearity analysis

**Fig. 2:**
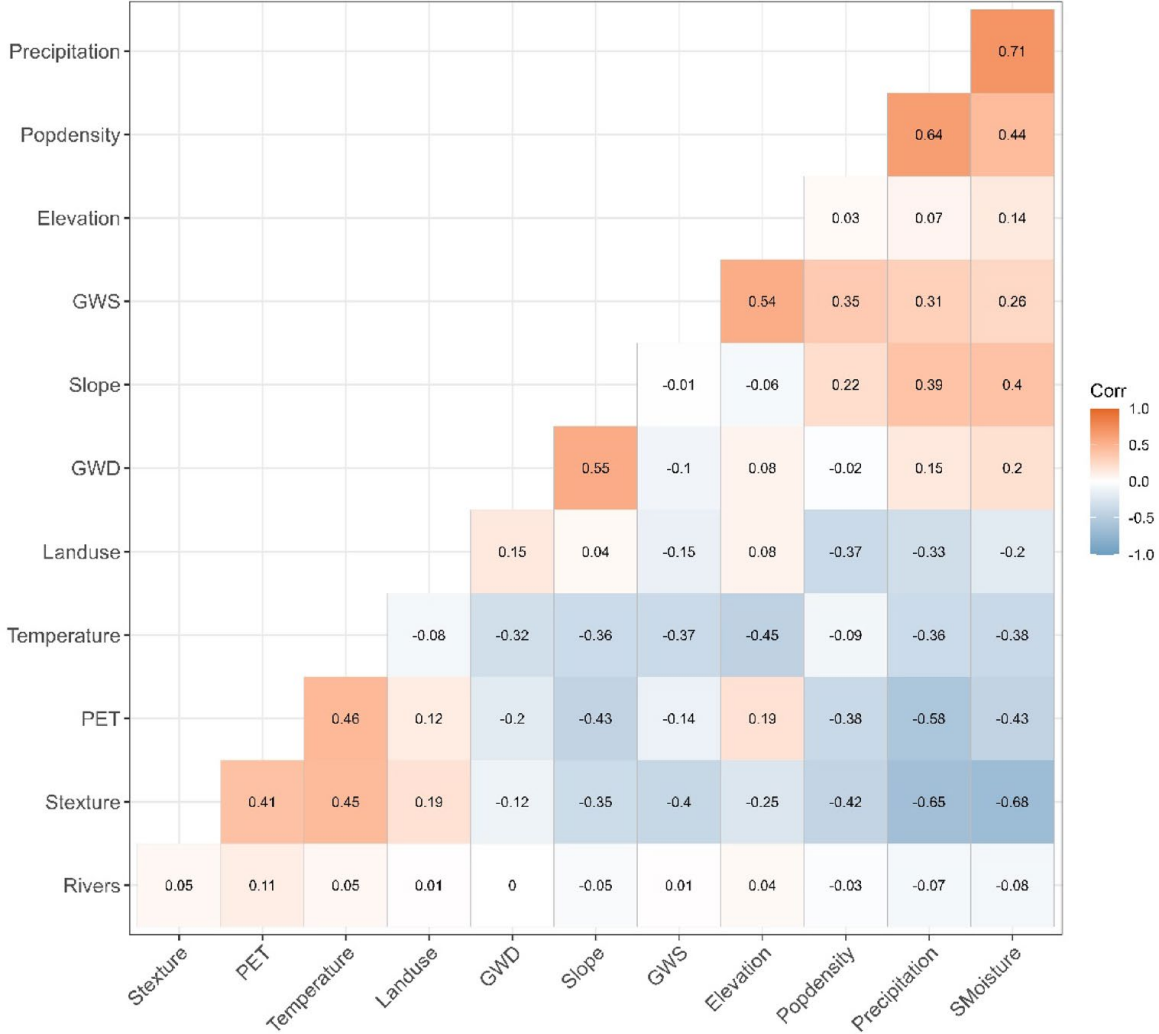
Correlation matrix for multicollinearity test for all the predictor variables.

The multicollinearity analysis showed no collinearity between the variables selected based on the set threshold (r <|0.8|). Variables that were related but not collinear were precipitation and soil moisture (r =|0.71|) and between precipitation and soil texture (r =|0.65|) (Fig. 2). The other related variables were soil moisture and texture (r =|0.68|). Regardless of the relationship, these variables had, they were all retained in the modelling process since they are relevant in determining the distribution of irrigation suitability.

### Model performance

**Fig. 3:**
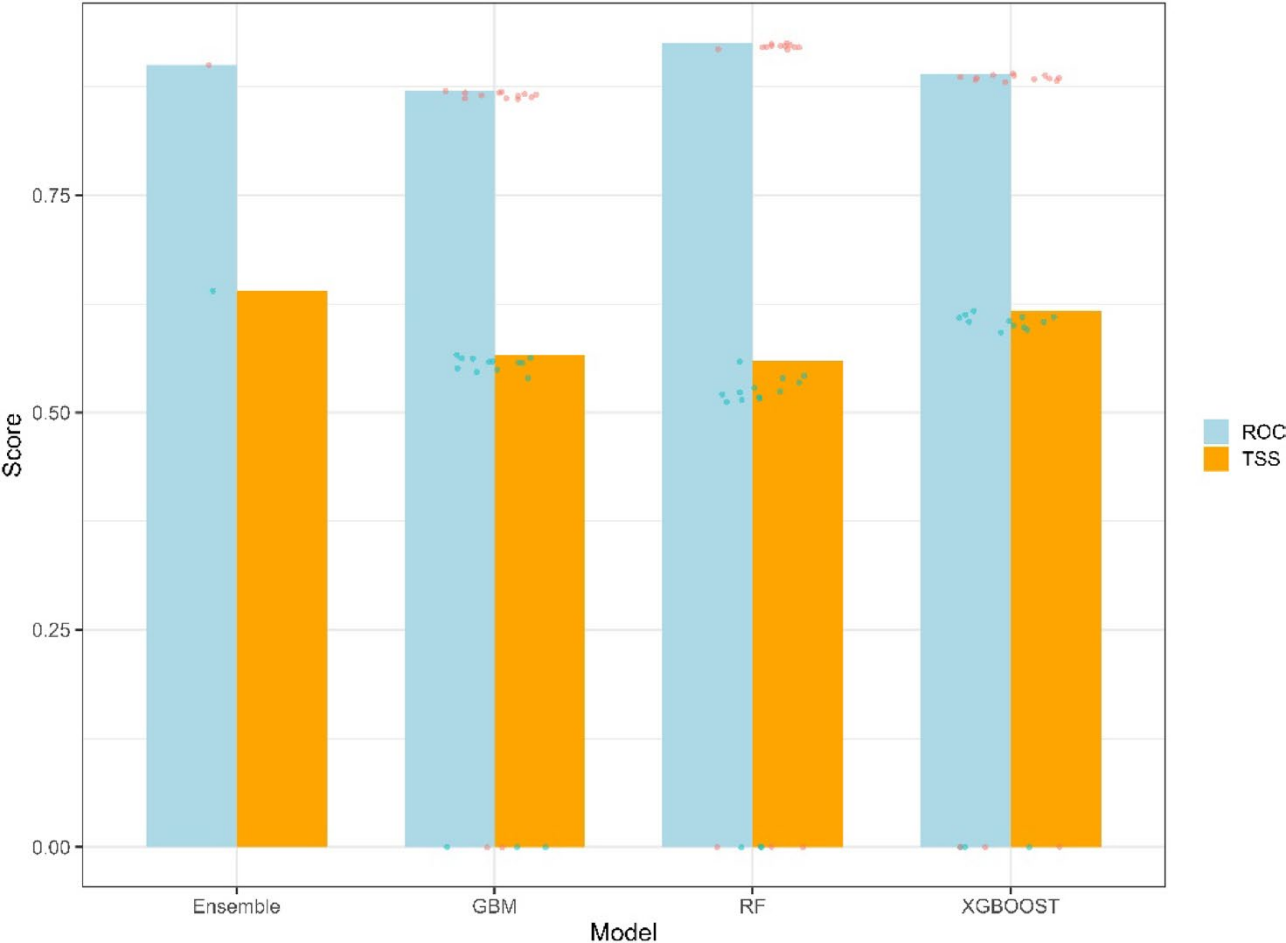
True Skill Statistic (TSS) and Area under the receiver Operating Curve (AUC) for the GBM, RF, XGBOOST, and ensemble models used for predicting irrigation suitability.

The performance of the candidate models used to predict irrigation suitability in South Africa exhibited considerable variability. Based on evaluation metrics, including the True Skill Statistic (TSS) and the Area Under the Curve (AUC), the XGBoost model demonstrated the highest accuracy (TSS = 0.60, AUC = 0.89), followed by the Gradient Boosting Machine (GBM) model (TSS = 0.57, AUC = 0.87) and the Random Forest model (TSS = 0.56, AUC = 0.93) (Fig. 3). Notably, the ensemble model, which integrated the strengths of the individual candidate models, outperformed all models, achieving the highest predictive performance with a TSS of 0.66 and an AUC of 0.90.

#### Variable importance and contribution

**Fig. 4:**
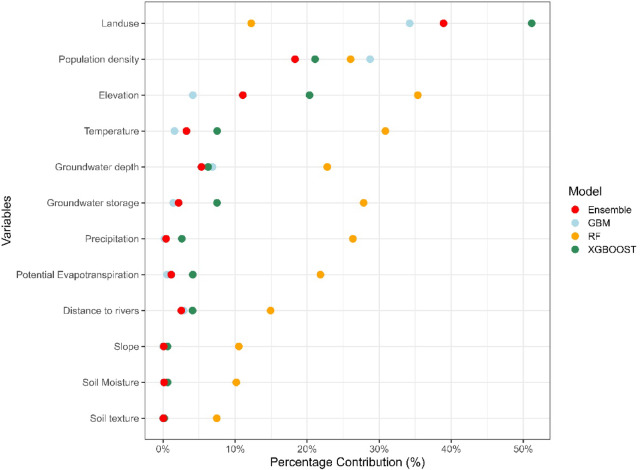
Variable contribution of predictor variables for different models, including GBM, RF, XGBOOST, and ensemble.

The variable importance analysis of predictor variables across models (GBM, RF, XGBoost, and Ensemble) revealed distinct patterns of influence (Fig. 4). For the GBM, land use emerged as the most significant contributor to model performance (34.2%), followed by population density (28.7%), while other variables contributed minimally. Similarly, in the XGBoost model, land use exhibited the highest contribution (51.2%), exceeding all other variables. In contrast, the RF model was dominated by elevation (35.4%) and temperature (30.8%), with precipitation (26.3%) and population density (26.0%) also playing significant roles. The ensemble model’s performance was primarily driven by land use (38.9%), followed by population density (18.3%) and elevation (11.1%). This balanced approach highlights the ensemble model’s ability to integrate the strengths of individual models, emphasising the combined influence of landscape and demographic factors.

**Fig. 5:**
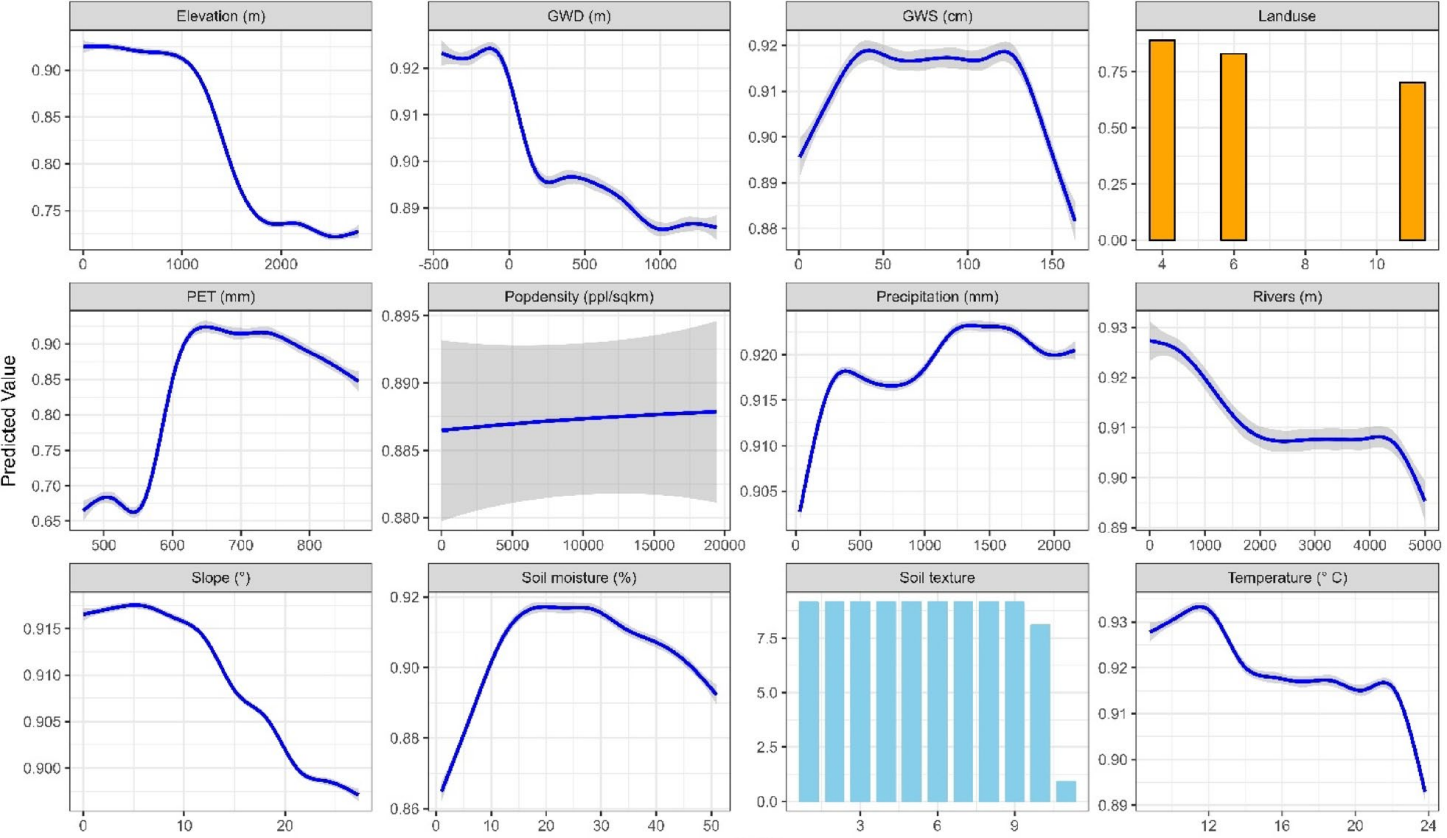
Response curves from the ensemble model show the effect of each predictor variable on irrigation suitability. For landuse, the values 4, 6 and 11-represents croplands, bare ground and rangelands respectively. For soil texture 1–11 represents clay, silty clay, sandy clay, clay loam, silty clay loam, sandy clay loam, loam, silt loam, sandy loam, silt and loamy sand.

The response curves from the ensemble model provided additional insights into irrigation suitability (Fig. 5). Suitability was highest in areas characterised by low elevation (0–1000 m), low slope (0–10°), higher precipitation (1000–2000 mm), and potential evapotranspiration (PET) (600–750 mm). Specific land use types, including existing croplands and bare ground were highly suitable for irrigation. Regarding groundwater variables, irrigation suitability improved with groundwater depth (GWD) values between 0 and 100 m from the surface and groundwater storage (GWS) ranging from 40 to 120 m. The effect of population density on irrigation suitability remained constant across varying densities, suggesting limited sensitivity to this factor in the context of irrigation potential. Areas with silt and loamy sand were also unsuitable for irrigation.

#### Distribution of irrigation suitability

**Fig. 6:**
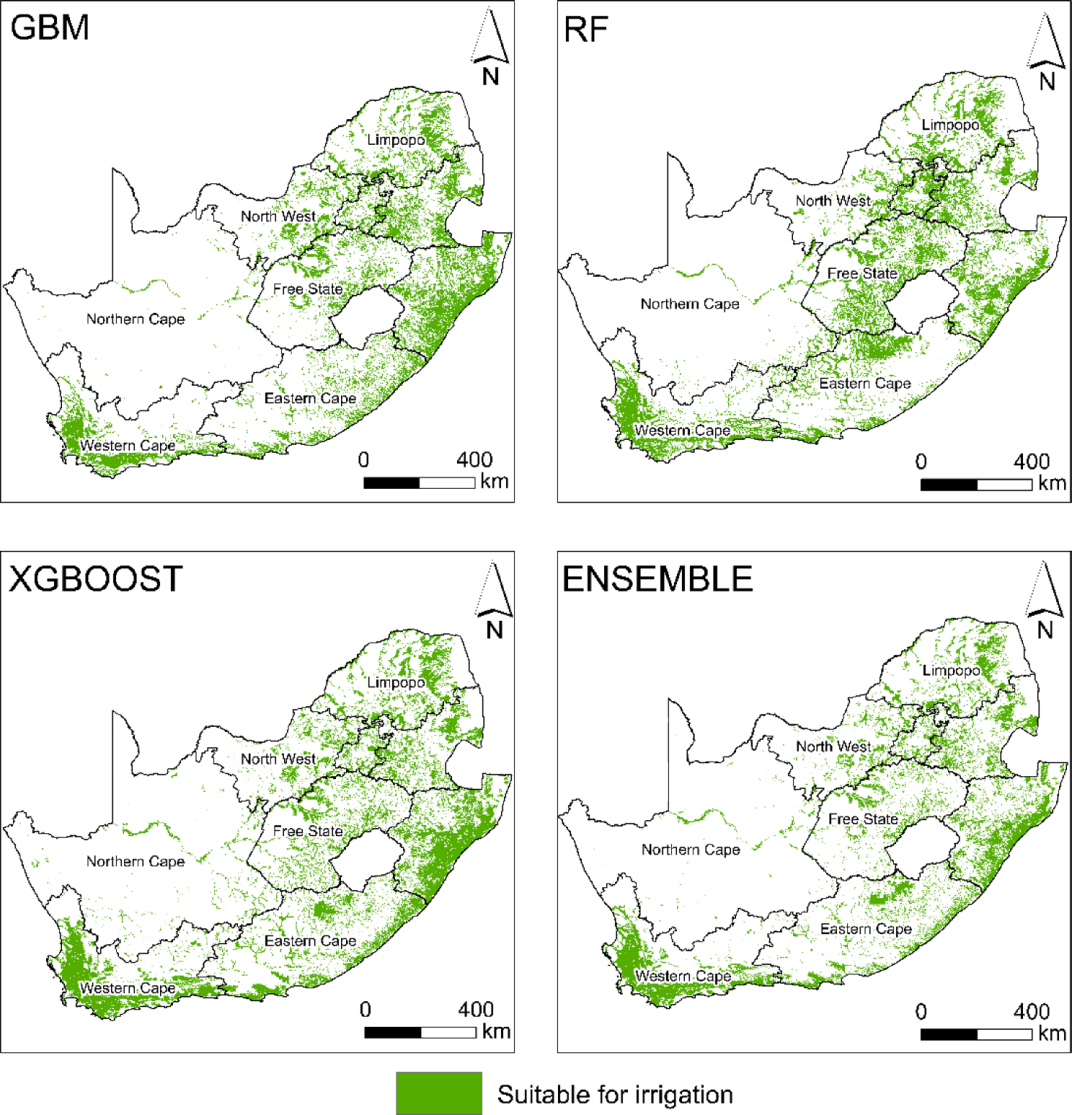
Distribution of irrigation suitability based on GBM, RF, XGBOOST, and ensemble models.

The spatial distribution of irrigation suitability varied significantly across South Africa, depending on the model applied in the study (Fig. 6). Specifically, the GBM predicted an irrigation suitability area of 232,760 km^2^ while RF estimated a larger suitable area of 258,488 km^2^. XGBoost identified the highest suitable area at 272,472 km^2^. Ensemble modelling, which integrated the strengths of individual models, yielded the most conservative estimate with an appropriate area of 202,945 km^2^.

The ensemble model’s suitability estimates for irrigation revealed notable spatial variation across South Africa’s provinces. The Northern Cape emerged as the province with the largest suitable area, covering 88,309 km^2^ (6.8%), followed by the Western Cape with 71,564 km^2^ (5.7%), presenting significant opportunities for irrigation expansion. The Eastern Cape accounted for 62,500 km^2^ (4.8%) of the national suitable area, while KwaZulu-Natal contributed 58,462 km^2^ (4.6%). In Limpopo, 41,500 km^2^ (3.3%) were identified as ideal, while the Free State and Mpumalanga provinces provided 30,421 km^2^ (2.4%) and 33,053 km^2^ (2.6%), respectively. The Northwest province had a smaller suitable area of 24,047 km^2^ (1.9%), while the highly urbanised Gauteng province exhibited the smallest suitability area, with 8,510 km^2^ (0.7%).

## Discussion

Irrigation suitability is pivotal for enhancing food security by pinpointing areas apt for agricultural expansion, thereby optimising productivity. This study highlights the utility of ensemble modelling in assessing irrigation potential across South Africa.

The ensemble model demonstrated a slightly superior predictive accuracy when compared to individual models such as RF and XGBoost. This superiority stems from the ensemble modelling’s ability to amalgamate highly predictive components of individual models into a cohesive “consensus” model, thereby enhancing overall accuracy^112,113^. This aggregation mitigates the limitations of individual models, reducing overfitting and improving generalisation to unseen data^31^. This approach is particularly beneficial for regions such as South Africa, characterised by complex and varied topography and climate, influencing irrigation suitability across different areas^114^. The complex interplay of these environmental variables, often exhibiting non-linear relationships with irrigation suitability, necessitates advanced modelling techniques^114,115^. Ensemble models, with their ability to capture these non-linearities^116^, provide a robust tool for accurate suitability assessments. For instance, the impact of elevation on irrigation suitability might be mediated by temperature and precipitation patterns, a complex relationship that an ensemble model can effectively learn^117,118^. Similarly, soil properties such as texture and drainage can interact with slope to influence water retention and, thus, irrigation needs^119,120^. Ensemble methods can disentangle these intricate relationships and provide more realistic representations of irrigation suitability. While ensemble modelling has been extensively and successfully applied in other domains, such as species distribution modelling (e.g., Mpakairi, Ndaimani^89^) and groundwater potential mapping (e.g., Pham, Jaafari^121^), its growing application in irrigation suitability assessments underscores the expanding utility of machine learning algorithms in agricultural planning.

The ensemble model identified land use as the primary driver of irrigation suitability, particularly in regions with existing croplands, and bare ground. The presence of existing croplands suggests inherently arable land with established soil properties, such as adequate fertility, organic matter content, and favourable structure, which can support successful irrigation farming^122,123^. These areas are often characterized by historical agricultural activities that have contributed to the development of resilient cropping systems, improved soil moisture retention, and a landscape already modified for cultivation^18,59,66^. Furthermore, the identification of bare ground as a key determinant highlights the potential for land conversion into irrigated agricultural systems. While bare ground may indicate land that is currently fallow, shallow soils, rocky or underutilized, it can also represent marginal lands with variable soil properties that could be enhanced through appropriate irrigation and soil management techniques^13,17^. Previous studies have noted that strategic irrigation infrastructure, combined with sustainable land-use planning, can transform such areas into productive agricultural zones, particularly in semi-arid and arid environments where water availability is the primary constraint^14,124^. These findings underscore the importance of prioritising areas with established agricultural activities for optimising productivity and minimising the substantial costs associated with developing entirely new irrigation systems^125,126^.

These findings align with global studies indicating that while biophysical factors such as climate and topography influence irrigation suitability, land use and land cover (LULC) often serve as stronger predictors (e.g., Teshome and Halefom^127^, Mandal, Dolui^128^ and^35^). Existing agricultural land use patterns reflect a complex interplay of environmental conditions, historical land management practices, and socio-economic factors^35,73^. As a result, areas with established agricultural activities often provide more favourable conditions for irrigation development than those lacking such integrated factors.

### Provincial agriculture expansion and development

The provincial analysis conducted in this study revealed significant regional variations in irrigation suitability across South Africa. Notably, the Northern Cape province had the most considerable area suitable for irrigation expansion, accounting for 6.8% of the nationally suitable area. This highlights the Northern Cape’s substantial potential for agricultural development through strategic irrigation investments^129,130^. Beyond the Northern Cape, the Western Cape, Eastern Cape, and KwaZulu-Natal provinces also exhibited considerable potential for irrigation expansion, collectively representing a significant portion of the country’s suitable areas^131–133^. These findings underscore the geographically dispersed opportunities for enhancing agricultural productivity owing to the complex geographic and climatic diversity.

The substantial irrigation potential identified in the Eastern Cape and KwaZulu-Natal is particularly noteworthy given the historical context of crop abandonment in these provinces^134,135^. Crop abandonment, often driven by drought, lack of water resources, and socio-economic constraints, has negatively impacted agricultural productivity and rural livelihoods in these regions^135,136^. Strategic irrigation development in these provinces offers a promising pathway to revitalise agricultural activities, mitigate the risk of future crop abandonment by providing a more reliable water supply, and significantly enhance overall agricultural productivity^136,137^. Expanding irrigation infrastructure in the Western Cape, a province already renowned for its export-oriented agricultural sector^138,139^, presents an opportunity to boost productivity further and drive economic growth. This expansion can lead to increased export revenues, job creation, and overall economic development within the province.

### Implications to sustainable agriculture planning

The findings of this study have significant implications for sustainable agricultural planning in South Africa. Agriculture is a major employer, particularly in rural areas, and its growth is widely recognised as a crucial driver of poverty reduction and improved livelihoods (e.g.,^140,141^. By strategically focusing irrigation investments on regions with high potential for agricultural expansion, policymakers can stimulate economic activity, create much-needed employment opportunities, and promote sustainable economic development^142,143^. Targeted irrigation development can catalyse rural revitalisation, enhances farm incomes and creates downstream employment opportunities in processing, packaging, and transportation^143,144^.

Integrating advanced modelling techniques, such as the ensemble modelling approach employed in this study, with comprehensive environmental and infrastructural data underscores the growing potential of data-driven approaches in addressing complex agricultural challenges in diverse and rapidly changing environments^31,112^. By harnessing the power of data analytics and machine learning, we can move beyond traditional, often less precise methods of agricultural planning and towards a more informed, efficient, and sustainable approach^18,23,116^. This allows for more targeted interventions, optimised resource allocation, and a more productive and resilient agricultural sector that can contribute to economic growth and food security in South Africa. Future development of these models could incorporate real-time data on weather conditions, soil moisture, and crop health to enhance their predictive capabilities and provide farmers with even more precise and actionable information.

### Limitations of the study and future research directions

While this study provides valuable insights into irrigation suitability across South Africa, it is essential to acknowledge certain limitations that should be considered when interpreting the results. Firstly, it is crucial to recognise that irrigation suitability, as modelled in this study, does not directly translate to high yield potential. A highly suitable area might underperform if inappropriate crop varieties are selected. While suitable areas indicate a biophysical capacity for irrigation, actual agricultural productivity is influenced by a complex interplay of factors beyond the scope of this analysis^18,124^. These include, but are not limited to, soil health and fertility, specific crop selection and management practices, pest and disease pressure, access to inputs (e.g., fertilisers, seeds), and market access for agricultural products^20^. Therefore, while the identified suitable areas represent promising opportunities for irrigation development, realising their full yield potential requires a holistic approach that addresses these additional factors.

Secondly, this study adopted a generalised approach to assessing irrigation suitability, which does not explicitly account for the diverse and often crop-specific irrigation needs. Different crops have varying water requirements, and a generalised suitability assessment may not accurately reflect the optimal irrigation strategies for specific agricultural systems^35,114^. For instance, a region deemed suitable for irrigation, in general, might be highly appropriate for a water-intensive crop such rice but only marginally suitable for a drought-tolerant crop like sorghum^35^. Consequently, the results presented here should be interpreted cautiously when applied to specific agricultural planning scenarios. The suitability maps provide a valuable overview of regional potential, but further, more granular analysis is required for crop-specific irrigation planning.

Future research should address these limitations and build upon the findings of this study by incorporating several key improvements. Using an independent dataset for spatial validation should be considered where feasible, and integrating crop-specific analyses into suitability models would provide a more nuanced understanding of irrigation potential and allow for more targeted agricultural planning. This would involve considering the specific water requirements of different crops and tailoring irrigation strategies accordingly. Furthermore, incorporating socio-economic and policy factors into suitability models is crucial for a more holistic and actionable assessment. Factors such as land ownership patterns, access to credit and markets, and existing agricultural policies can significantly influence the feasibility and sustainability of irrigation development^123^. Integrating these factors would provide a more realistic picture of irrigation potential and inform policy decisions that support sustainable agricultural expansion.

Finally, examining the potential impacts of climate change on water availability and future irrigation needs is essential for long-term agricultural planning. Climate change projections suggest that many regions may experience altered precipitation patterns, increased temperatures, and more frequent droughts^30,59^, impacting water availability and irrigation requirements. For instance, the Western Cape province in South Africa endured its worst drought during 2015/16, prompting the provincial government to implement the ‘Day Zero’ water conservation measures^145^. Integrating climate change scenarios into suitability models would allow for a more robust assessment of long-term irrigation potential and inform adaptation strategies to ensure sustainable agricultural production in a changing climate. Future studies can provide more comprehensive, actionable, and forward-looking insights to guide sustainable agricultural expansion and development in South Africa by addressing these gaps.

## Conclusion

Identifying areas for irrigation expansion is imperative for optimising agriculture productivity and enhancing food security. This study sought to map areas suitable for irrigation across South Africa using ensemble modelling. The results showed that irrigation suitability was more probable in areas with existing croplands. The results also showed that the Northern Cape province had the most considerable area suitable for irrigation expansion. The findings of this study have significant implications for sustainable agricultural planning and allow investments in regions with high potential for agricultural expansion. Policymakers can utilise these results to stimulate economic activity, create much-needed employment opportunities, reduce poverty and promote sustainable economic development. Future research could explore the specific crop types best suited for irrigation in each province and the potential for integrating small-scale farmers into larger irrigation schemes to maximise the benefits of these investments.

## Data Availability

The data used in this research is freely available online and can also be obtained upon request from the corresponding author.
